# On the Excito Motory System of Marshall Hall

**Published:** 1854-08

**Authors:** 


*On the Excito Motory System of Marshall Hall.* By J. G. Davey, M. D.

The author of this paper endeavors to show that the excito motory phenomena, as explained by Dr. M. Hall rests on a basis which even *he* up to this time has failed to recognize. His object is to demonstrate that the true spinal functions stand in no need of the distinct set of nervous fibres—the exodic and eisodic nerves—which Dr. Hall describes and Mr. Grainger endeavored to demonstrate with the scalpel; but that the sensory and motor spinal nerves are of themselves sufficient to create all the phenomena with which Dr. Hall has made the profession so fully acquainted.

Dr. Davey contended that the excito motory system can be roused into action when the spinal marrow has been separated from the brain, or even in the case of an acephalous monster born without a brain; but even more; that precisely the same phenomena have occurred in monsters born without either a brain or a *spinal marrow;* and quotes Sir Gilbert Blane to support him in this statement. He declares that the reflex actions, properly so called, are nothing more than an amplification of the ordinary spinal functions; an additional exciting cause either from within or without.

An effort is then made to prove that the *organic system*, with the solar ganglion for its centre is the great mainspring of all the phenomena of life; whether they relate to the organic or animal functions; whether to the brain, spinal marrow or any other individual part of the whole organism. He declares that the ganglia of the sympathetic system are in existence in the embryo anterior to the cerebro spinal organism, and that the medulla spinalis, as well as the brain; the heart and other internal viscera; all exercise their several functions in virtue of the power derived fron the ganglionic system. He denies the correctness of Dr. Hall's experiments and states that the removal or destruction of the sympathetic system in the frog has always been found fatal to the true spinal phenomena. The motions so obviously perceptible are nothing more than retained nervous influence, and continue no longer than the peculiar contractile irritability remains. The following passage is quoted from Dr. Hall's book on the "Nervous system" to strengthen his position. "I must add that as the irritability of the internal organs depends much on the ganglionic system, so that of the muscles of the limbs may partly depend on the latter system. Although I have observed this irritability to be greatly diminished by the removal of the influence of the spinal marrow, I think," he adds, "I never knew it to be completely annihilated under such circumstances."

Again, Dr. Davey says, the function of respiration is under the control of the spinal system, and when that ceases, it is an evidence that the spinal cord has lost its functions. How, then, can we account for the fact that the uterus has been able tö expel a child after respiration had entirely ceased, which he declares to have occurred, unless upon the supposition that the ganglionic system still continued to give vitality and muscular power to the organ ?

He therefore believes—

"1. That the more complicated anatomy of the spinal cord, suggested by Dr. M. Hall, and sought for by Mr. Grainger, has not yet been realized.

2. That the mere sensory and motor nerves or roots are themselves sufficient to the integrity of all those phenomena called excito-motory, which emanate from the spinal cord; or, in other words, that there is no necessity for any distinct division of the nervous (spinal) system, for the production of the excito-motory phenomena.

3. That the exercise of the functions of the spinal cord is due to the peculiar influence of the ganglionic nervous system, exerted on its organism.

4. That both the external and internal excito-motory phenomena, are alike dependent on the vital stimulus imparted by the great sympathetic nerve, and, in the former instance, through the instrumentality or medium of the medulla spinalis.

5. That such is the relationship between the great nervous centres, viz., the brain, spinal cord, and the solar ganglion, that the second, (viz., the spinal cord,) being at one time under the dominion of the first, *i. e.* the brain, and at another time under that of the third, *i. e.* the solar ganglion, and being thus intermediate, will now manifest a cerebral or voluntary power (and this the result of consciousness, and, of course, volition,) and then (the antecedent circumstances being dissimilar) display one of an involuntary, automatic, or instinctive, or excito-motory character; but this only, of course, when the organic nervous apparatus is in the ascendant, and the brain, comparatively quiescent—or, in other words, when sensation (spinal) and motion, and not consciousness and volition (cerebral attributes) are in operation, if I may be allowed so to express myself.

6. That the sympathetic nerve is endowed with a specific and independent power, to which both the brain and spinal cord, and with them, of course, their functions are subordinate."— *Virginia Med. and Surg. Journal.*

*Researches on the Sympathetic Nerve.* By M. Claude Bernard.

When the great sympathic nerve is divided in the neck, or its ganglia excised, the results which follow are, retraction of the eye-ball, contraction of the pupil, redness of the conjunctiva, and the projection of the cartilage of the third eye-lid. These phenomena were observed first by Pourfour du Petit (1727,) and afterwards by Dupuy, Brachet, and John Reid. In 1846 M. Biffie noticed that by galvanising the cephalic end of the divided nerve, the pupil which was contracted in consequence of the section of the nerve, would become again dilated.

The experiments of MM. Budge and Waller proved that the influence transmitted by the sympathetic was derived from that portion of the spinal cord situate between the last cervical and sixth dorsal vertebra, which they named the cilio-spinal region.

M. Bernard, announced to the Academy of Sciences, in March, 1852, his important discovery of the great increase of animal heat, and of sensibility on that side of the head on which the sympathetic was divided; and also ascertained that upon the application of galvanism to the upper end of the nerve this animal heat would disappear. Budge referred this increase of heat to the cilio-spinal region. Waller and Brown Séquard explained the increased heat, and vascularity by the paralysis of the arterial system caused by section of the nerve; Brown Séquard claiming to have been the first to show that galvanism of the sympathetic diminished the temperature and produced contraction of the arteries.

M. Bernard then proceeded to prove by a series of experiments:

1st. That a division of the sensory nerves in addition to the abolition of sensation also diminished the temperature of the part.

2. The division of the motor nerves destroyed the power of motion and also diminished the animal heat of the parts.

3. The destruction of the sympathetic producing neither a loss of motion or sensation, greatly increased the temperature.

This augmentation of the animal heat is therefore a property peculiar to the sympathetic nerve. The elevation of temperature begins instantaneously with the section of the nerve, and amounts to 4° or 5° centigrade. The excess of calorification upon a section of the nerve lasts in rabbits from sixteen to eighteen days, and in dogs about two months; but after extirpation of the ganglia, its effect have remained very intense a year and a half after the removal of the ganglia.

Upon the application of galvanism to the divided nerve, reverse phenomena are produced. The pupil dilates, the vascularization of the parts disappear, and their temperature falls.

"I have only wished," says the author, "in this communication, to establish one point in the complex history of the great spmpathetic, namely, that the section of the filaments or ganglions of that nerve possess constantly the privilege of increasing the calorification of the parts to which it is distributed. These phenomena are only the exaggeration of what naturally takes place in the production of animal heat. In giving the means of increasing the production of heat and localizing it in external parts which are easily observed, I had thought of rendering more accessible to our means of research the study of that important function which is still so little known, but which cannot be investigated elsewhere than in the greater or less activity of the chemical metamorphoses which the blood undergoes in the living textures under special influences of the nervous system."—*Gaz. Med.*—*Ib.*

---

For the above important researches, the Academy of Sciences awarded to M. Bernard the annual prize of experimental physiology for the year 1853.—ED. VA. MED. AND SURG. JOURNAL.

---

*Adulteration of Drugs.*—The following resolution was adopted at the last meeting of the American Medical Association:

" *Resolved*—That in the Secretary to the Treasury's recommendation to Congress, to abolish or materially modify the duty on such crude drugs not producible in this country as are used in the laboratories of the country in the manufacture of chemicals, we recognise a wise provision for the further protection of the profession and the community at large, from impure and sophisticated medicines."

